# Supplementary material for: Residential Proximity to Urban Play Spaces and Childhood Overweight and Obesity in Barcelona, Spain: A Population-Based Longitudinal Study
Source: Int J Environ Res Public Health. 2022 Oct 21;19(20):13676. doi: 10.3390/ijerph192013676 (PMC9603004; doi:10.3390/ijerph192013676)
Supplement: Supplementary file 1 [file ijerph-19-13676-s001.zip › ijerph-1966670-supplementary.pdf]

Supplemental material

Residential proximity to urban play spaces and childhood overweight and obesity in Barcelona, Spain: a population-based longitudinal study

Nacho Sánchez-Valdivia [1,2,*], Carmen Pérez-del-Pulgar [1,2,3,4], Jeroen de Bont [5], Isabelle Anguelovski [1,2,6], Antonio López-Gay [7,8], Andrea Pistillo [9], Margarita Triguero-Mas [10, 1,2,*,†] and Talita Duarte-Salles [9,†]

Author List and Affiliations:

[1] Barcelona Lab for Urban Environmental Justice and Sustainability, Institute of Environmental Science and Technology (ICTA), Universitat Autònoma de Barcelona (UAB), 08003 Barcelona, Spain

[2] Hospital del Mar Medical Research Institute (IMIM), Carrer Doctor Aiguader, 88, 08003 Barcelona, Spain

[3] Helmholtz Centre for Environmental Research—UFZ Department Environmental Politics, 04318 Leipzig, Germany

[4] Department for Political Science, Friedrich-Schiller-University, *07737* Jena, Germany

[5] Institute of Environmental Medicine, Karolinska Institute, 171 77 Stockholm, Sweden

[6] ICREA (Institució Catalana de Recerca i Estudis Avançats), 08010 Barcelona, Spain

[7] Department of Geography, Universitat Autònoma de Barcelona (UAB), 08193 Barcelona, Spain

[8] Center for Demographic Studies, 08193 Bellaterra, Spain

[9] Fundació Institut Universitari per a la Recerca a l'Atenció Primària de Salut Jordi Gol i Gurina (IDIAPJGol), 08007 Barcelona, Spain

[10] Mariana Arcaya's Research Lab, Department of Urban Studies and Planning, Massachusetts Institute of Technology, 77 Massachusetts Ave, Cambridge, MA 02139, USA

***** Correspondence: nachis23@gmail.com (Nacho Sánchez-Valdivia); mtrigueromas@gmail.com (Margarita Triguero-Mas).

† Joint last authorship.

Supplemental material content

- Figure S1. Flowchart of included population.
- Figure S2. Example of children's follow-up.
- Figure S3. Directed acyclic graph (DAG) for childhood overweight and obesity and play spaces exposure.
- Figure S4. Estimated linearity between play space exposures and childhood overweight or obesity.
- Figure S5. Spearman correlations matrix between the outdoor residential play spaces indicators (overall, green and diversity of them).
- Table S1. Population description and residential proximity to play spaces distribution and bivariate analysis by sex (N =75608).
- Table S2. Bivariate analysis of childhood overweight developed during follow-up by area SES (quintiles of deprivation) (N = 22215).
- Table S3. Association between residential proximity to play spaces and the development of childhood obesity (excluding overweight) (N = 83975).
- Table S4. Adjusted association between residential proximity to play spaces and the development of childhood obesity (excluding overweight) by area SES (quintiles of deprivation) (N = 83975).
- Table S5. Adjusted association between residential proximity to play spaces and the development of childhood overweight by non-movers during follow-up period (N = 57654).
- Table S6. Adjusted association between residential proximity to play spaces and the development of childhood overweight adjusting for Spanish and non-Spanish nationality (N= 75608).
- Table S7. Adjusted association between residential proximity to play spaces and the development of childhood overweight by sex adjusting for additional maternal characteristics (maternal nationality and maternal BMI) (N = 14645).

Figure S1. Flowchart of included population.

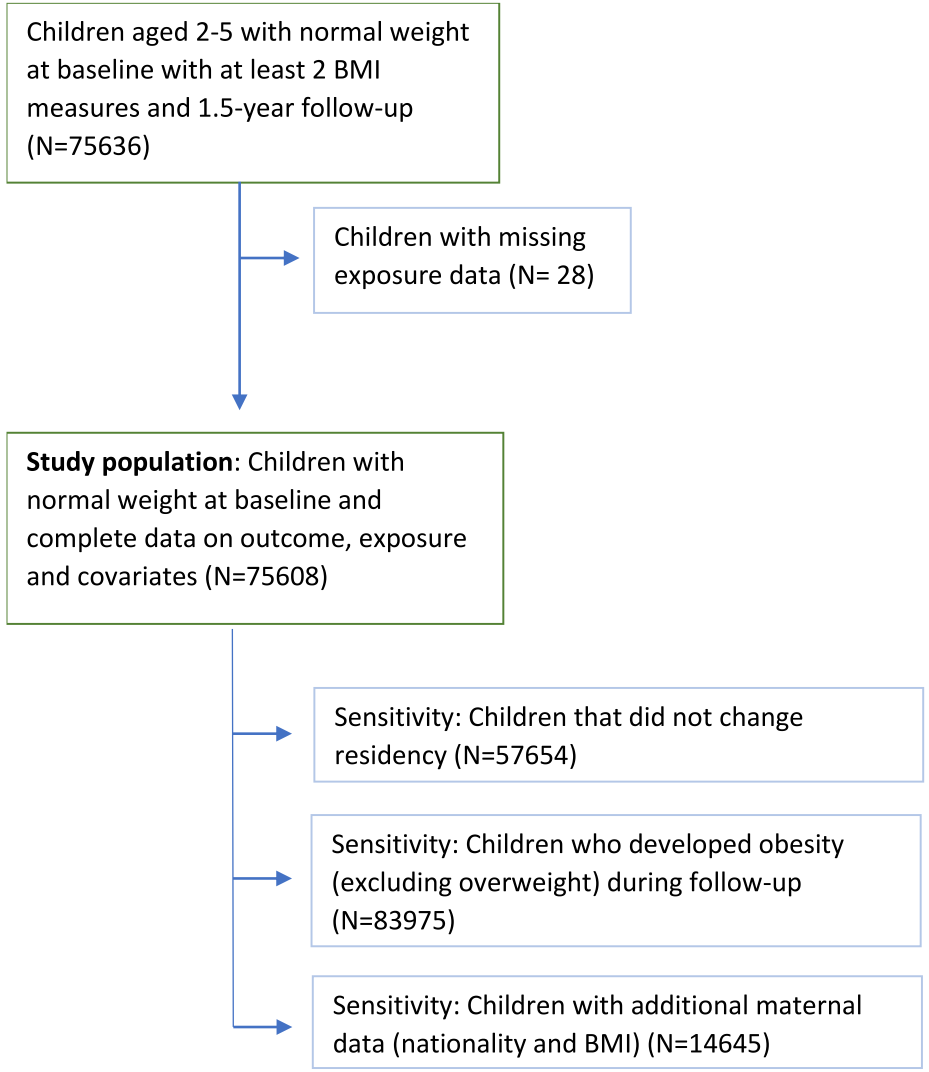

Figure S2. Example of children's follow-up.

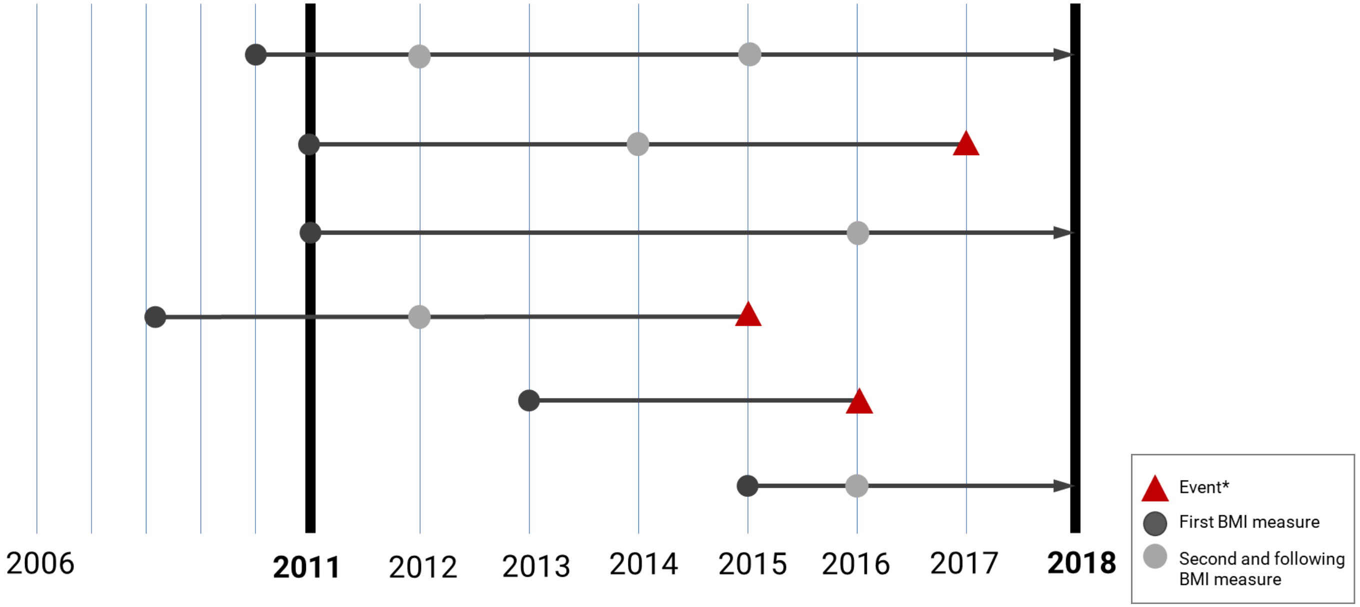

*When individuals reach 15 years old, become overweight or obese, transfer-out of SIDIAP, or die.

Figure S3. Directed acyclic graph (DAG) for childhood overweight and obesity and play spaces exposure.

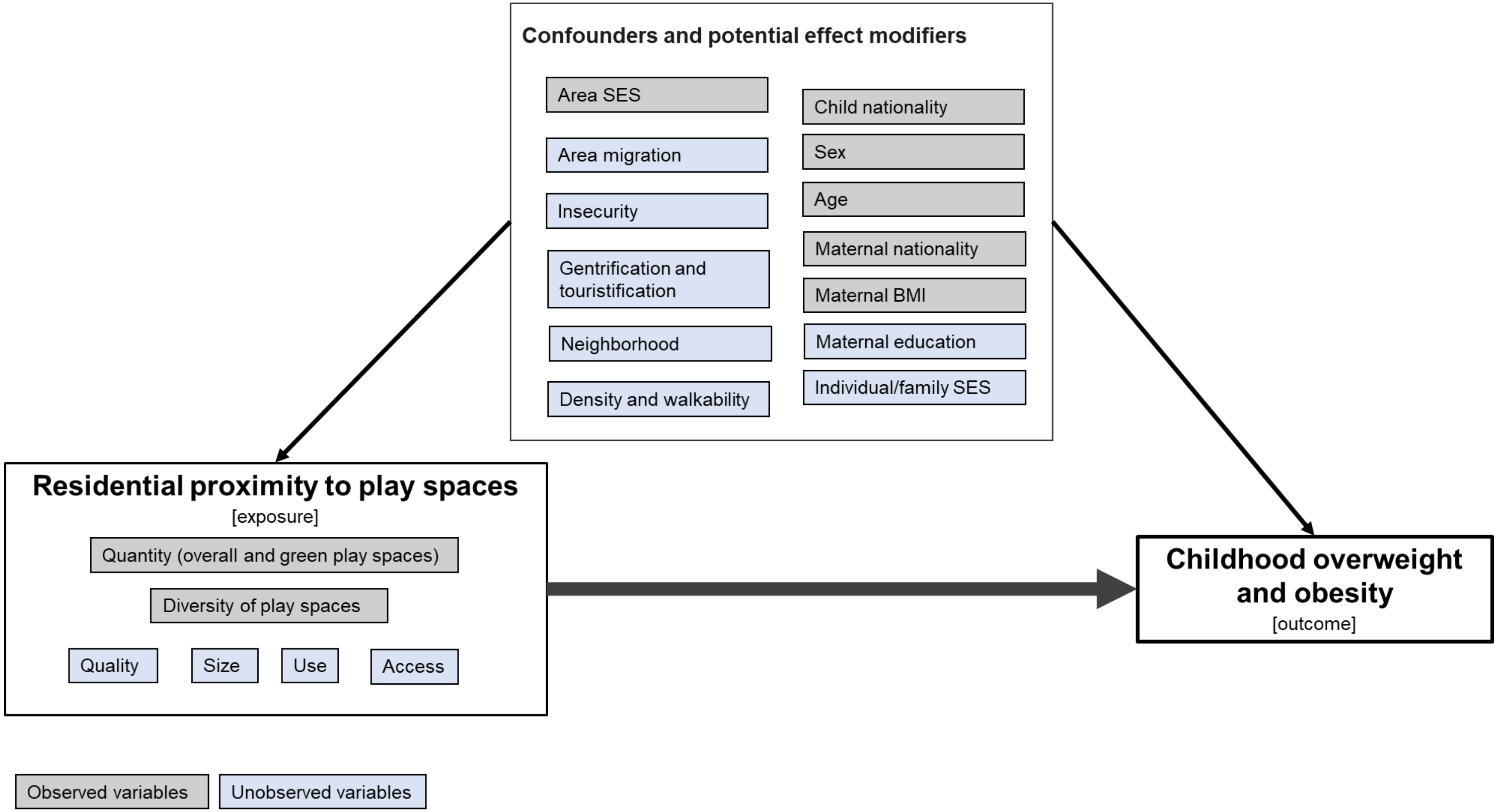

Source: self-made

Figure S4. Estimated linearity between play space exposures and childhood overweight or obesity.

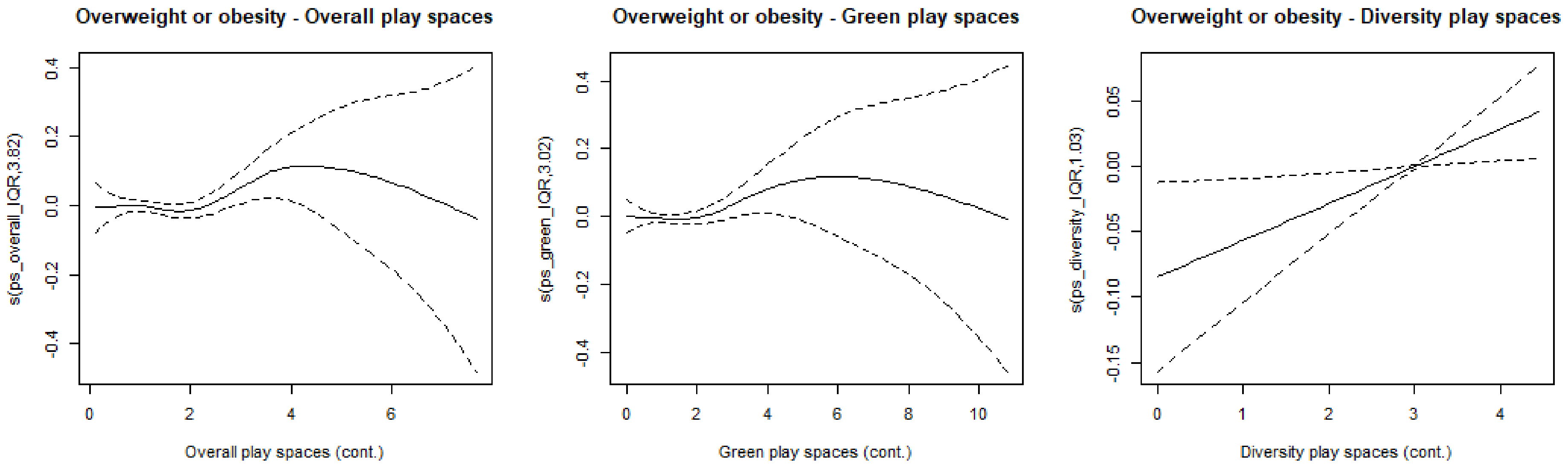

Note: We examined the linear relationship between each play space exposure indicator and childhood overweight or obesity using generalized additive models (GAM). These Models are adjusted by child's age at baseline (categorical), population aged 2-14 living in the children's residential area, child's sex, child nationality and area deprivation index.

Figure S5. Spearman correlations matrix between the outdoor residential play spaces indicators (overall, green and diversity of them).

	Overall	Green
**Green**	0.83*	
**Diversity**	0.01*	0.13*

* p-value < 0.05.

Table S1. Population description and residential proximity to play spaces distribution and bivariate analysis by sex (N=75608).

	Boys			Girls		
	**Overall**	**Remained normal weight during follow-up**	**Developed overweight**	**Overall**	**Remained normal weight during follow-up**	**Developed overweight**
	**N= 38775 (51.28%)**	**N= 26870 (69.30%)**	**N=11905 (30.70%)**	**N= 36833 (48.72%)**	**N=26523 (72.08%)**	**N=10310 (28.00%)**
**Age baseline**, years median (p25; p75)	2.11 (2.06; 2.63)	2.12 (2.06; 2.62)	2.11 (2.06; 2.69)	2.12 (2.06; 2.68)	2.12 (2.06; 2.70)	2.11 (2.05; 2.64)
**Age at case**, years median (p25; p75)	6.33 (6.04; 8.30)	-	6.33 (6.04; 8.30)	6.31 (6.05; 8.19)	-	6.31 (6.05; 8.19)
**Time of follow-up**, years median (p25; p75)	5.80 (3.89; 8.47)	6.57 (4.19; 9.68)	4.10 (3.32; 5.98)	5,65 (3.95; 8.77)	6.80 (4.27; 9.92)	4.09 (3.35; 5.86)
**Children's nationality**, N %						
Global north	36206 (93.37%)	25132 (93.53%)	11074 (93.02%)	34432 (93.48)	24860 (93.73%)	9572 (92.84%)
Global south	2569 (6.63%)	1738 (6.47%)	831 (6.98%)	2401 (6.52%)	1663 (6.27%)	738 (7.16%)
**Area deprivation level (quintiles),** N (%)						
First (least deprived)	7762 (20.02%)	5825 (21.68%)	1937 (16.27%)	7362 (19.99)	5718 (21.56%)	1644 (15.95%)
Second	7731 (19.94%)	5580 (20.77%)	2151 (18.07%)	7387 (20.06)	5465 (20.60%)	1922 (18.64%)
Third	7740 (19.96%)	5319 (19.80%)	2421 (20.34%)	7386 (20.05)	5343 (20.14%)	2043 (19.82%)
Fourth	7756 (20.00%)	5121 (19.06%)	2635 (22.13%)	7364 (19.99)	5101 (19.23%)	2263 (21.95%)
Fifth (most deprived)	7786 (20.08%)	5025 (18.70%)	2761 (23.19%)	7334 (12.91)	4896 (18.46%)	2438 (23.65%)
**Residential proximity to play spaces,** median (p25; p75)						
Overall	12.00 (8.00; 17.00)	12.00 (8.00; 17.00)	12.00 (8.00; 17.00)	12.00 (8.00; 17.00)	12.00 (8.00; 17.00)	12.00 (8.00; 17.00)
Green	6.00 (4.00; 9.00)	6.00 (4.00; 9.00)	6.00 (4.00; 9.00)	6.00 (4.00; 9.00)	6.00 (4.00; 9.00)	6.00 (4.00; 9.00)
Diversity	0.93 (0.72; 1.01)	0.93 (0.72; 1.01)	0.93 (0.74; 1.01)	0.93 (0.72; 1.01)	0.92 (0.72; 1.01)	0.93 (0.72; 1.01)

p25= 25th percentile, p75= 75th percentile. For continuous variables, values are median and p25; p75. For categorical variables, absolute number and percentage.

Table S2. Bivariate analysis of childhood overweight developed during follow-up by area SES (quintiles of deprivation) (N = 22215).

	**Deprivation level (quintiles)**				
	**First (least deprived)** **N= 3584**	**Second** **N= 4071**	**Third** **N= 4467**	**Fourth** **N= 4893**	**Fifth (most deprived)** **N= 5200**
**Girls**, N (%)	1644 (45.87%)*	1923 (47.24%)*	2043 (45.74 %)*	2262 (46.23%)*	2438 (46.88%)*
**Age baseline**, years median (p25; p75)	2.13 (2.06; 3.00)	2.11 (2.06; 2.66)	22.11 (2.06; 2.62)	2.11 (2.05; 2.60)	2.11 (2.05; 2.57)
**Age at case**, years median (p25; p75)	6.35 (6.05; 8.29)	6.32 (6.04; 8.24)	6.33 (6.05; 8.26)	6.35 (6.03; 8.24)	6.29 (6.04; 8.21)
**Time of follow-up**, years median (p25; p75)	4.10 (3.29; 5.90)*	4.10 (3.35; 5.93)*	4.1 (3.37; 5.96)*	4.1 (3.30; 5.98)*	4.09 (3.32; 5.87)*
**Children's nationality**, N %					
Global north	3444 (96.09%)*	3875 (95.19%)*	4173 (93.42%)	4503 (92.03%)	4651 (89.44%)*
Global south	140 (3.91%)*	196 (4.81%)*	294 (6.58%)	390 (7.97%)	549 (10.56%)*
**Residential proximity to play spaces** median (p25; p75)					
Overall	11.00 (8.00; 16.00)	13.00 (9.00; 18.00)*	13.00 (9.00; 18.00)	12.00 (8.00; 17.00)*	11.00 (7.00; 16.00)
Green	6.00 (4.00; 8.00)	6.00 (4.00; 10.00)	7.00 (4.00; 10.00)	6.00 (4.00; 9.00)	6.00 (4.00; 9.00)
Diversity	0.94 (0.69; 1.03)	0.92 (0.69; 1.02)	0.92 (0.78; 1.00)	0.94 (0.80; 1.01)*	0.94 (0.69; 1.01)

p25= 25th percentile, p75= 75th percentile. For continuous variables, values are median and p25; p75. For categorical variables, absolute number and percentage.
* p-value < 0.05. Chi-Square Test for categorical variables, Student's test for parametric distributions and Mann-Whitney's U test and Kruskal-Wallis's test for non-parametric.

Table S3. Association between residential proximity to play spaces and the development of childhood obesity (excluding overweight) (N = 83975).

	All	Boys	Girls
	HR (95% CI)*	HR (95% CI)*	HR (95% CI)*
**Overall play spaces**	1.01 (0.98; 1.03)	1.00 (0.97; 1.04)	1.01 (0.97; 1.04)
**Green play spaces**	1.01 (0.98; 1.02)	1.00 (0.97; 1.03)	0.99 (0.96; 1.03)
**Diversity play spaces**	1.01 (0.98; 1.04)	1.00 (0.96; 1.03)	1.01 (0.97; 1.08)

Note: Hazard ratios (HR) reported by one IQR increase in outdoor play space indicators within 300m from children's census tracts of residence. Analyses are made separately for each (3) residential proximity to outdoor play space indicators

*These models are adjusted child's age at baseline (categorical) in the strata statement, population aged 2-14 living in the children's residential area, child's sex, child nationality and area deprivation index.

Table S4. Adjusted association between residential proximity to play spaces and the development of childhood obesity (excluding overweight) by area SES (quintiles of deprivation) (N = 83975).

	First (least deprived)	Second	Third	Fourth	Fifth (most deprived)
	HR (95% CI)*	HR (95% CI)*	HR (95% CI)*	HR (95% CI)*	HR (95% CI)*
**Overall play spaces**	1.02 (0.95; 1.10)	1.02 (0.96; 1.09)	0.97 (0.92; 1.03)	0.98 (0.93; 1.03)	1.02 (0.98; 1.07)
**Green play spaces**	0.99 (0.92; 1.06)	0.99 (0.95; 1.05)	1.00 (0.95; 1.05)	0.98 (0.94; 1.03)	1.00 (0.97; 1.04)
**Diversity play spaces**	0.96 (0.88; 1.03)	0.97(0.91; 1.03)	1.02 (0.96; 1.09)	1.01 (0.95; 1.07)	1.06 (0.99; 1.12)

Note: Hazard ratios (HR) reported by one IQR increase in outdoor play space indicators within 300m from children's census tracts of residence. Analyses are made separately for each (3) residential proximity to outdoor play space indicators

*These models are adjusted child's age at baseline (categorical) in the strata statement, population aged 2-14 living in the children's residential area, child's sex, child nationality and area deprivation index.

Table S5. Adjusted association between residential proximity to play spaces and the development of childhood overweight by non-movers during follow-up period (N = 57654).

	All	Boys	Girls
	HR (95% CI)	HR (95% CI)	HR (95% CI)
**Overall play spaces**	1.03 (1.01; 1.05)	1.02 (1.00; 1.05)	1.03 (1.00; 1.06)
**Green play spaces**	1.02 (1.00; 1.04)	1.02 (1.00; 1.04)	1.02 (0.99; 1.04)
**Diversity play spaces**	1.02 (1.00; 1.05)	1.02 (0.98; 1.05)	1.03 (1.00; 1.06)

Note: Hazard ratios (HR) reported by one IQR increase in outdoor play space indicators within 300m from children's census tracts of residence. Analyses are made separately for each (3) residential proximity to outdoor play space indicators

*These models are adjusted child's age at baseline (categorical) in the strata statement, population aged 2-14 living in the children's residential area, child's sex, child nationality and area deprivation index.

Table S6. Adjusted association between residential proximity to play spaces and the development of childhood overweight adjusting for Spanish and non-Spanish nationality (N= 75608).

	All	Boys	Girls
	HR (95% CI)*	HR (95% CI)*	HR (95% CI)*
**Overall play spaces**	1.02 (1.00; 1.03)	1.02 (0.99; 1.04)	1.01 (0.99; 1.04)
**Green play spaces**	1.01 (1.00; 1.04)	1.02 (1.00; 1.04)	1.01 (0.99; 1.03)
**Diversity play spaces**	1.02 (1.00; 1.04)	1.01 (0.98; 1.04)	1.03 (1.00; 1.06)

Note: Hazard ratios (HR) reported by one IQR increase in outdoor play space indicators within 300m from children's census tracts of residence. Analyses are made separately for each (3) residential proximity to outdoor play space indicators

*These models are adjusted child's age at baseline (categorical) in the strata statement, population aged 2-14 living in the children's residential area, child's sex, child nationality and area deprivation index.

Table S7. Adjusted association between residential proximity to play spaces and the development of childhood overweight by sex adjusting for additional maternal characteristics (maternal nationality and maternal BMI) (N = 14645).

	All	Boys	Girls
	**HR (95% CI)***	**HR (95% CI)***	**HR (95% CI)***
**Overall play spaces**	1.00 (0.96; 1.04)	1.00 (0.95; 1.06)	0.99 (0.94; 1.05)
**Green play spaces**	1.01 (0.97; 1.04)	1.00 (0.96; 1.06)	1.00 (0.96; 1.05)
**Diversity play spaces**	1.00 (0.95; 1.05)	0.96 (0.90; 1.02)	1.05 (0.98; 1.12)

Note: Hazard ratios (HR) reported by one IQR increase in outdoor play space indicators within 300m from children's census tracts of residence. Analyses are made separately for each (3) residential proximity to outdoor play space indicators

*These models are adjusted child's age at baseline (categorical) in the strata statement, population aged 2-14 living in the children's residential area, child's sex, child nationality, area deprivation index, maternal nationality and maternal BMI (categorized as normal weight, overweight or obesity).
